# Night work during pregnancy and risk of cryptorchidism among male offspring: A Danish nationwide register‐based cohort study

**DOI:** 10.1111/andr.70055

**Published:** 2025-05-07

**Authors:** Charlotte Bertelsen, Luise Mølenberg Begtrup, Paula E. C. Hammer, Jens Peter Ellekilde Bonde, Anne Helene Garde, Ina Olmer Specht, Johnni Hansen, Esben Flachs Meulengracht, Camilla Sandal Sejbæk

**Affiliations:** ^1^ Department of Occupational and Environmental Medicine Bispebjerg and Frederiksberg University Hospital Bispebjerg Denmark; ^2^ Department of Public Health University of Copenhagen Copenhagen Denmark; ^3^ National Research Centre for the Working Environment Copenhagen Denmark; ^4^ The Parker Institute Bispebjerg and Frederiksberg University Hospital Frederiksberg Denmark; ^5^ Section for General Practice, Department of Public Health University of Copenhagen Copenhagen Denmark; ^6^ Danish Cancer Institute, Danish Cancer Society Copenhagen Denmark

**Keywords:** cryptorchidism, maternal exposure, night shift, night work, occupation, pregnancy

## Abstract

**Aim:**

The aim was to investigate the association between night work during pregnancy and the risk of having a male offspring with cryptorchidism. Furthermore, we explored if the risk of cryptorchidism increased based on trimester‐specific night work (gestational weeks 1–12 and 13–22) by sensitivity analyses.

**Methods:**

This register‐based cohort study was based on detailed objective working hour data for all employees in the five Danish regions (primarily hospital employees) between 2007 and 2015, retrieved from the Danish Working Hour Database (DWDH). Information on pregnancies and covariates was identified by linking DWDH with the Danish Medical Birth Register. Diagnoses of cryptorchidism were obtained from the Danish National Patient Register. We used logistic regression to investigate the association between different dimensions of night work during the first 32 pregnancy weeks and cryptorchidism. The adjusted models included maternal age, body mass index, socioeconomic position, and maternal smoking.

**Results:**

The final cohort consisted of 12,915 singleton pregnancies in 11,404 women (primarily nurses), who worked at least one night shift during the first 32 pregnancy weeks. None of the dimensions of night work was associated with an increased risk of having offspring with cryptorchidism compared to day workers. We found the same tendency in the trimester‐specific analyses.

**Conclusions:**

We found no increased odds among women working night shifts in healthcare during pregnancy and giving birth to male offspring with cryptorchidism. Future studies investigating night work in occupations other than healthcare are needed to rule out a potential association.

## 1 Introduction

Cryptorchidism (unilaterally or bilaterally undescended testes) is one of the most common malformations in boys and occurs in 3.2% of newborn boys in Denmark.^1^ Cryptorchidism is associated with an increased risk of testicular cancer and infertility in adulthood.^2^

Even though, several pathogenetic mechanisms and the influence of environmental factors in relation to cryptorchidism have been described,^4^ the etiology of cryptorchidism remains unknown in most cases. In specific, testicular descent involves two hormonally controled phases, which are believed to occur prenatally in the transabdominal phase at gestational weeks 8–15 and the inguinoscrotal phase at gestational weeks 25–35.^3^ Pathogenetic mechanisms, such as the hormonal environment during these two phases, seem to be crucial for testicular descent.^4^, ^5^ Insulin‐like factor 3 (INSL3) regulates the first phase and androgens regulate the second phase. The hormones are regulated and produced by Leydig cells.^6^ Disruption of these two phases, as well as factors interfering with the Leydig cells, may increase the risk of giving birth to a male offspring with cryptorchidism.^5^ Lower levels of INSL3 have been found in the cord blood of male offspring with cryptorchidism compared to male offspring with normally descended testes.^7^

An experimental rat study suggested a ‘programming window’ for the reproductive tract masculinization corresponding to gestational weeks 8–14 in humans. In rats, disruption of the hormonal environment during this window leads to cryptorchidism.^8^ A Danish case–control study of 421 cases with cryptorchidism and 425 controls of singleton male offspring showed that the concentration of INSL3 in amniotic fluid samples during gestational weeks 13–16 was higher in cryptorchidism cases compared to controls. Two sensitive time windows have been suggested. First, one window was suggested in gestational weeks 13–16 due to testicular descent.^9^ Second, a Danish cohort study hypothesized that normal testicular descent, regulated by Leydig cell biosynthesis in the fetal testis, occurs during the first trimester of pregnancy (gestational weeks 1–12).^10^

Epidemiological studies have suggested that external exposures and mothers’ occupational environments, including night work during pregnancy, may affect the hormones controling testicular descent.^11^, ^12^ A plausible mechanism, that night work affects the risk of cryptorchidism in the male offspring, could be exposure to light during night work. The light disturbs circadian rhythms and may influence the tightly coordinated hormone levels resulting in lower melatonin production.^12^ Melatonin plays a key role in regulating Leydig cell activity.^13^ A study showed that pregnant women working night shifts had lower melatonin levels compared to pregnant day workers. The authors suggested that light exposure during night work influenced the change in melatonin levels.^14^

Only one previous study has investigated the association between night work during pregnancy and cryptorchidism. This Japanese study used self‐reported data from 51,316 pregnancies to evaluate working hours and found no association between night work during pregnancy and having male offspring with cryptorchidism.^11^ However, the use of self‐reported data introduced a risk of misclassification, potentially leading to an underestimation of an association in this study.

We aimed to investigate, if night work during pregnancy was associated with the risk of having a male offspring with cryptorchidism using objective and detailed register‐based payroll data from a large Danish population of health professionals. Furthermore, our detailed data allowed for sensitivity analyses, investigating whether night work during the first (weeks 1–12) or second (weeks 13–22) trimester of pregnancy was associated with an increased risk of cryptorchidism.

## 2 Materials and methods

We conducted a prospective register‐based cohort study using information from three Danish national registries, which were linked at individual level using the unique personal identification number (PIN) all citizens have in Denmark. We used data from the Danish Working Hour Database (DWHD). This database contains detailed administrative payroll data with daily information on individual working hours for all employees in public hospitals in the five Danish administrative regions from 2007 to 2021.^15^ In this study, we had information from January 2007 to December 2015. All pregnant employees were identified by linking DWHD with data from the Danish Medical Birth Register (DMBR).^16^ Information on cryptorchidism was obtained from the Danish National Patient Registry (DNPR).

### 2.1 Study population

**FIGURE 1:**
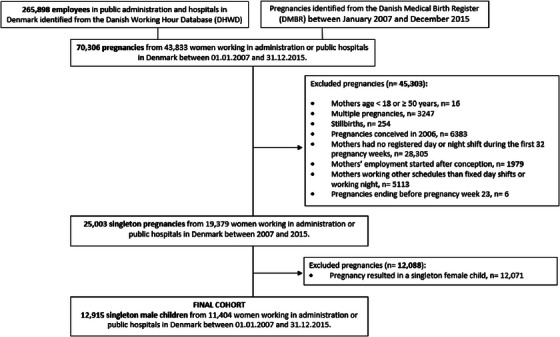
Flowchart for identification of the study population.

Women from DWDH, who gave birth to at least one child between 2007 and 2015, were identified (*n* = 43,833). We excluded women if they: were < 18 or ≥ 50 years of age (*n* = 16), had a multiple pregnancy (*n* = 3247), had stillbirth (*n* = 254), conceived the pregnancy before 2007 (prior to the first year of registration in DWDH) (*n* = 6383), were not registered as employed in DHWD at the time of conception (*n* = 1979), worked schedules other than fixed day or night shifts during the first 32 pregnancy weeks (*n* = 5113), had a pregnancy that terminated before pregnancy week 23 (*n* = 6), had no registered day or night shifts during the first 32 weeks of pregnancy (*n* = 28,305), or gave birth to a female child (*n* = 12,071). The final study population consisted of pregnancies where the offspring was a live‐born, singleton male child (*n* = 12,915) (Figure 1).

### 2.2 Exposure assessment

Information on working hours was obtained from DWHD and included precise details on shift start and end times of each working shift. Night shifts were defined as shifts with ≥3 working hours between 23:00 and 06:00 h, while day shifts included shifts as ≥3 h between 06:00 and 21:00 h. Furthermore, evening shifts were defined as ≥3 working hours between 21:00 and 02:00 h, and early morning shifts were to start between 03:00 and 06:00 h, in accordance with Härmä et al.^17^ Night workers were defined as women working at least one night shift during the first 32 gestational weeks.

In line with previous studies based on the same cohort,^18^, ^19^, ^20^, ^21^ we examined night work by four dimensions: (1) number of night shifts, (2) duration of night shifts, (3) consecutive night shifts, and (4) quick returns after night shifts. These four dimensions were summarized for the first 32 weeks of pregnancy. The number of night shifts was categorized into 1–7, 8–16, 17–24, and ≥25 shifts. Duration of night shift was defined as ≤8, 9–12, and > 12 h or more night shifts. Consecutive night shifts were grouped into 1, 2–3, and ≥4 night shifts. Number of quick returns after night shifts were grouped into none, 1–8, and ≥9 night shifts, and defined as a recovery period of ≤28 h after a night shift. We used a cut‐off at 32 weeks of pregnancy because most female employees in the Danish administrative regions were entitled to maternity leave beginning at 33 weeks of pregnancy.^22^

### 2.3 Outcome assessment

Information on cryptorchidism was obtained from the DNPR, which covers all Danish public and private hospitals and contains information on hospital contacts, inpatient diagnoses, and surgical procedures (23). Cryptorchism was defined using the International Classification of Disease (ICD) 10 codes: Q53, Q53.1, Q53.1A, Q53.2, Q53.2A, Q53.9, and/or surgical procedure codes for cryptorchidism: KKFH00, KKFH01, KKFH10 (Nordic Classification of Surgical Procedures).

### 2.4 Potential confounders

Confounders included in the analyses were chosen a priori based on a directed acyclic graph (DAG)^24^ (Figure S1). We included maternal age (continuous variable), pre‐pregnancy body mass index (BMI; < 18.5, 18.5–24.9, 25.0–29.9, and ≥30 kg/m^2^), and smoking status during pregnancy (non‐smoker, stopped during pregnancy, current smoker). All recorded at the first antenatal visit by a midwife or doctor and registered in the DMBR.^16^ Socioeconomic position (SEP; low, middle, high) was based on job titles, which were converted into two versions of the Danish version of International Standard Classification of Occupations (DISCO‐88 and DISCO‐08).^25^, ^26^ Information on maternal pre‐pregnancy diabetes was retrieved from DNPR, and job title was obtained from DWHD.^15^, ^23^

### 2.5 Statistics

We investigated the risk of having a male offspring with cryptorchidism in relation to four dimensions of night work during the first 32 gestational weeks using logistic regression, considering both crude and adjusted models. The results from the models are presented as odds ratios (OR) with 95% confidence interval (CI). The adjusted model included maternal age, pre‐pregnancy BMI, SEP, and smoking. Only 17 women were registered with diabetes prior to pregnancy and was therefore not included in the adjusted models. All analyses were performed using day workers as the reference group. Day workers were defined as women who worked at least one day shift and had no night, evening, or early morning shifts during the first 32 gestational weeks of pregnancy. Additionally, we conducted sensitivity analyses focusing on sensitive time windows for testicular descent. We investigated night shifts during the first trimester (weeks 1–12) and the second trimester (weeks 13–22) and the risk of having a male offspring with cryptorchidism, respectively. Due to the smaller sample size when investigating exposures in the first or the second trimester separately, the four dimensions of night shifts were grouped into fewer categories. The statistical analyses were conducted in SAS 9.4 (SAS Institute Inc, Cary, NC, USA).

## 3 Results

The cohort consisted of 11,404 unique women with 12,915 singleton pregnancies. Of these, 6992 women (61.2%) were night workers (worked at least one night shift during the first 32 gestational weeks). On average, night workers had 15 night shifts during the first 32 gestational weeks, and only 45 women worked permanent night shifts during their pregnancy. Night and day workers were very similar on demographic characteristics. The mean age of the study population was 32 years. Most women had a BMI within the normal range (65%) and were non‐smokers (94.4%). The two groups differed regarding SEP, with a higher proportion of night workers having medium SEP (70.0% vs. 55.9%) and a lower proportion having low SEP (17.8% vs. 6.4%) compared to day workers. Night workers were primarily nurses (63.7%), whereas day workers were mostly administrative personnel and other occupations unrelated to healthcare (14.7%). The prevalence of cryptorchidism was 3.20% among night workers and 3.18% among day workers (Table 1).

**TABLE 1:** Characteristics of the women of 12,915 singleton pregnancies and their male offspring born among 11,404 healthcare workers in Denmark 2007–2015.

Characteristics	All pregnancies *n* = 12,915 (11,404 women)		Night work^a^‐ pregnancies *n* = 7882 (6992 women)^c^		Day work^b^‐ pregnancies *n* = 5033 (4563 women)^c^	
**Maternal age at conception**, mean (SD)	31.6	(3.91)	31.2	(3.80)	32.3	(4.00)
**Pre‐pregnancy BMI** ^d^, *n* (%)						
Underweight	403	(3.1)	229	(2.9)	174	(3.5)
Normal weight	8394	(65.0)	5157	(65.4)	3237	(64.3)
Overweight	2499	(19.3)	1502	(19.1)	997	(19.8)
Obese	1145	(8.9)	691	(8.8)	454	(9.0)
Missing, *n* (%)	474	(3.7)	303	(3.8)	171	(3.4)
**Smoking during pregnancy**, *n* (%)						
Non‐smoker	12,194	(94.4)	7466	(94.7)	4728	(93.9)
Stopped during pregnancy	191	(1.5)	127	(1.6)	64	(1.3)
Current smoker	325	(2.5)	167	(2.1)	158	(3.1)
Missing, *n* (%)	205	(1.6)	122	(1.6)	83	(1.7)
**Socioeconomic position**, *n* (%)						
High	3104	(24.0)	1836	(23.3)	1268	(25.2)
Medium	8331	(64.5)	5518	(70.0)	2813	(55.9)
Low	1401	(10.9)	504	(6.4)	897	(17.8)
Missing, *n* (%)	79	(0.6)	24	(0.3)	55	(1.1)
**Profession**, *n* (%)						
Nurse	5756	(44.6)	5018	(63.7)	738	(14.7)
Physician	1761	(13.6)	1377	(17.5)	384	(7.6)
Healthcare workers	1692	(13.1)	910	(11.5)	782	(15.5)
Medical secretary	977	(7.6)	48	(0.6)	929	(18.5)
Physio‐ and occupational therapist	736	(5.7)	25	(0.3)	711	(14.1)
Laboratory technician	567	(4.4)	204	(2.6)	363	(7.2)
Midwife	226	(1.8)	213	(2.7)	13	(0.3)
Other occupations	1200	(9.2)	87	(1.1)	1113	(22.1)
**Shifts during the first 32 gestational weeks**, mean (%)						
Day	78.84	(36.1)	61	(26.7)	107	(29.8)
Evening	8.2	(12.6)	13.4	(13.9)	–	
Night	8.9	(12.2)	14.6	(12.6)	–	
Early morning	0.02	(0.4)	0.03	(0.5)	–	
Mean weekly working hours	23.8	(7.1)	23.5	(7.1)	24.1	(7.1)
**Children**						
Cryptorchidism, *n* (%)	412	(3.19)	252	(3.20)	160	(3.18)

^a^ At least one night shift during the first 32 gestational weeks.

^b^ Only day workers with no registered night, evening, or early morning shift during the first 32 gestational weeks.

^c^ Some women had pregnancies with different working schedule status.

^d^ BMI, body mass index: underweight < 18.5 kg/m^2^, normal weight 18.5–24.9 kg/m^2^, overweight 25.0–29.9 kg/m^2^, obese >30 kg/m^2^.

None of the dimensions of night work during pregnancy (number of night shifts, consecutive night shifts, duration of night shifts, and quick returns) were associated with an increased risk of having a male offspring with cryptorchidism in either the crude or adjusted analyses (Table 2). The number of night shifts showed a tendency toward a dose‐dependent association with cryptorchidism for the first three categories; with increasing number of night shifts, we observed a higher odds of cryptorchidism in the offspring (17–24 night shifts; OR = 1.26, 95% CI 0.91–1.75). On the other hand, when the number of night shifts during pregnancy were 25 or more the odds of cryptorchidism was lower (OR = 0.90, 95% CI 0.61–‐1.31) compared to day workers. However, the ORs had limited precision with parameter values ranging from no effect to a considerable higher odds. The observed difference between the exposure and reference group was so small that we do not believe the statistically insignificant result raises any sample size or power issue. The sensitivity analyses (Table 3), of night work during the first (weeks 1–12) and second (weeks 13–22) trimester, respectively, showed results similar to the main analyses (Table 2).

**TABLE 2:** Odds ratios (OR) with 95% confidence intervals (CI) for cryptorchidism in male offspring by four dimensions of night work in 12,915 pregnancies among 11,404 healthcare workers in Denmark 2007–2015.

	All pregnancies *n* = 12,915		Number of cryptorchidism *n* = 412		Crude analysis		Adjusted analysis^a^	
	*n*	%	*n*	% ^b^	OR	95 % CI	OR	95 % CI
**Number of night shifts**								
Day work only^c^	5033	39.0	160	3.2	1	Ref	1	Ref
1–7	2665	20.6	84	3.2	0.99	(0.76–1.30)	1.03	(0.78–1.37)
8–16	2509	19.4	81	3.2	1.02	(0.77–1.33)	1.08	(0.81–1.43)
17–24	1429	11.1	52	3.6	1.15	(0.84–1.58)	1.26	(0.91–1.75)
≥ 25	1279	9.9	35	2.7	0.86	(0.59–1.24)	0.90	(0.61–1.31)
**Consecutive night shifts** ^e^								
Day work only^c^	5033	39.0	160	3.2	1	Ref	1	Ref
1	2628	20.4	87	3.3	1.04	(0.80–1.36)	1.12	(0.84–1.49)
2–3	3378	26.2	116	3.4	0.95	(0.75–1.22)	1.02	(0.79–1.32)
≥ 4	1876	14.4	49	2.6	1.08	(0.78–1.49)	1.09	(0.77–1.55)
**Duration of night shifts** ^f^								
Day work only^c^	5033	39.0	160	3.2	1	Ref	1	Ref
≤ 8 h	1800	13.9	62	3.4	1.09	(0.81–1.46)	1.10	(0.80–1.51)
9–12 h	2945	22.8	92	3.1	0.98	(0.76–1.27)	1.03	(0.78–1.37)
> 12 h	3137	24.3	98	3.1	0.98	(0.76–1.27)	1.08	(0.82–1.42)
**Quick returns after night shifts** ^d^, ^g^								
Day work only^c^	5033	39.0	160	3.2	1	Ref	1	Ref
None	550	4.2	13	2.4	0.74	(0.42–1.31)	0.74	(0.41–1.34)
1–8	4673	36.2	153	3.3	1.03	(0.82–1.29)	1.11	(0.87–1.41)
≥ 9	2659	20.6	86	3.2	1.02	(0.78–1.33)	1.06	(0.81–1.41)

^a^ Adjusted for maternal age, body mass index, smoking, socioeconomic position. *N* = 679 missing in the adjusted analyses due to missing information on covariates; pre‐pregnancy BMI, SES, and smoking.

^b^ Number divided with pregnancies in each group.

^c^ Only day workers with no registered night, evening, or early morning shift during the first 32 gestational weeks.

^d^ ≤ 28 h between a night shift and the next shift.

^e^ Only one single consecutive night shift; at least one spell of 2–3 consecutive night shifts; at least one spell of ≥ 4 consecutive night shifts.

^f^ Night shifts lasting ≤ 8 h; night shifts lasting 9–12 h; or night shifts lasting > 12 h.

^g^ At least one night shift and no quick returns; at least one night shift and 1–8 quick returns; at least one night shift and ≥ 9 quick returns.

**TABLE 3:** Odds ratios (OR) with 95% confidence intervals for cryptorchidism in male offspring by four different dimensions of night work during the first trimester (weeks 1–12) and second trimester (weeks 13–22) among healthcare workers in Denmark 2007–2015.

	All pregnancies		Number of cryptorchidism		Crude analysis		Adjusted analysis^a^	
	*n*	%	*n*	%	OR	95 % CI	OR	95 % CI
**Number of night shifts (1^st^ trimester)**								
Day work only^b^	5380	45.1	174	1.5	1	Ref	1	Ref
1–6 night shifts	2729	22.9	87	0.7	0.99	(0.76–1.28)	1.01	(0.77–1.33)
≥ 7	3826	32.0	133	1.1	1.08	(0.85–1.35)	1.15	(0.91–1.46)
**Number of night shifts (2^nd^ trimester)**								
Day work only^b^	6676	55.5	225	1.9	1	Ref	1	Ref
1–6 night shifts	2774	23.1	77	0.6	0.82	(0.63–1.07)	0.88	(0.67–1.15)
≥ 7	2574	21.4	86	0.7	0.99	(0.77–1.28)	1.05	(0.81–1.36)
**Consecutive night shifts** ^c^ **(1^st^ trimester)**								
Day work only^b^	3779	40.5	125	1.3	1	Ref	1	Ref
1	1694	18.2	52	0.6	0.93	(0.67–1.29)	0.98	(0.69–1.40)
2–3	2703	28.9	93	0.9	1.04	(0.79–1.37)	1.05	(0.79–1.41)
≥ 4	1154	12.4	36	0.4	0.94	(0.65–1.37)	0.93	(0.63–1.38)
**Consecutive night shifts** ^c^ **(2^nd^ trimester)**								
Day work only^b^	4846	52.0	166	1.8	1	Ref	1	Ref
1	1379	14.8	38	0.4	0.81	(0.56–1.15)	0.89	(0.60–1.29)
2–3	1972	21.1	66	0.7	0.98	(0.73–1.31)	1.02	(0.75–1.37)
≥ 4	1133	12.1	36	0.4	0.93	(0.64–134)	0.92	(0.63–1.35)
**Duration of night shifts** ^d^ **(1^st^ trimester)**								
Day work only^b^	5380	43.0	174	1.4	1	Ref	1	Ref
≤ 8 h	199	1.6	10	0.1	1.55	(0.81–2.98)	1.29	(0.62–2.66)
>8 h	6924	55.4	228	1.8	1.02	(0.83–1.24)	1.08	(0.87–1.33)
**Duration of night shifts** ^d^ **(2^nd^ trimester)**								
Day work only^b^	6676	53.4	225	1.8	1	Ref	1	Ref
≤ 8 h	230	1.8	9	0.1	1.16	(0.59–2.29)	1.25	(0.63–2.47)
>8 h	5597	44.8	178	1.4	0.94	(0.77–1.15)	0.99	(0.81–1.23)
**Quick returns after night shifts** ^e^ **(1^st^ trimester)**								
Day work only^b^	3225	41.0	103	1.3	1	Ref	1	Ref
None	282	3.6	7	0.1	0.78	(0.36–1.69)	0.70	(0.30–1.61)
1–2	895	11.4	29	0.4	1.02	(0.67–1.54)	0.93	(0.60–1.46)
≥ 3	3451	44.0	117	1.5	1.06	(0.81–1.39)	1.11	(0.83–1.47)
**Quick returns after night shifts** ^e^ **(2^nd^ trimester)**								
Day work only^b^	4130	52.6	137	1.7	1	Ref	1	Ref
None	187	2.4	3	0.1	0.48	(0.15–1.53)	0.52	(0.161.65)
1–2	512	6.5	12	0.2	0.71	(0.39–1.28)	0.69	(0.37–1.29)
≥ 3	3024	38.5	104	1.3	1.04	(0.80–1.34)	1.10	(0.84–1.44)

*Note*: First trimester: *n* = 11,935 pregnancies and second trimester: *n* = 12,024 pregnancies.

^a^ Adjusted for maternal age, body mass index, smoking, socioeconomic position. *N* = 503–1237 missing in the adjusted analyses due to missing information on covariates; pre‐pregnancy BMI, SES, and smoking.

^b^ Only day workers with no registered night shift, evening, or early morning shift.

^c^ Only one single consecutive night shift; at least one spell of 2–3 consecutive night shifts; at least one spell of ≥ 4 consecutive night shifts.

^d^ Night shifts lasting ≤ 8 h; night shifts lasting 9–12 h; or night shifts lasting > 12 h.

^e^ At least one night shift and no quick returns; at least one night shift and 1–8 quick returns; at least one night shift and ≥ 9 quick returns.

## 4 Discussion

In this large cohort study among healthcare professionals, we found no notable association between working night shifts during the first 32 gestational weeks and the odds of having a male offspring with cryptorchidism. The trimester‐specific sensitivity analyses supported the main findings.

Our findings were in line with the only other study investigating this association: a large Japanese study from 2020 based on self‐reported data from 51,316 pregnancies. Maternal exposure was assessed for women working in 14 different occupations including night shift. The researchers found no association between working night shifts in the first weeks of pregnancy (OR = 0.80, 95% CI 0.47–1.27) or mid‐to late pregnancy (OR = 0.63, 95% CI 0.30–1.16) and the risk of having a male offspring with cryptorchidism.^11^ However, the low prevalence of cryptorchidism (0.59%) in their study population and the use of self‐reported data on night work may have led to misclassification and an underestimation of a potential association.^11^ In this study, both the assessment of night shifts and the diagnosis of cryptorchidism were based on register data from an unselected cohort of healthcare workers, ensuring greater data accuracy and reliability.

Our study is, as far as we know, the first study to use objective and detailed prospective exposure data to investigate the association between night work during pregnancy and cryptorchidism in offspring. A major strength of this study was the detailed information on shift start and end time for all types of shifts during pregnancy. This reduces the risk of misclassification and makes it possible to investigate different dimensions of night work and potential dose‐response patterns. Furthermore, our outcome was based on national register data from DNPR, which has high validity and complete records on all patient diagnoses and surgeries across all Danish public hospitals.^27^ We therefore consider the risk of misclassification in our outcome data to be very low. In our cohort, we found an incidence of cryptorchidism of 3.2%, which aligns with the national incidence rate in Denmark.^1^

However, some limitations must be acknowledged. Women, who have previously experienced miscarriage or other adverse pregnancy outcomes, may avoid working night shifts, when they are trying to conceive compared to pregnant women without such experiences. This ‘healthy worker hire selection^32^’ could lead to null findings due to a downward‐biased risk estimate when day workers are used as the comparison group. Consequently, women in this cohort with a potential higher risk of having a child with cryptorchidism may have refrained from working night shifts before becoming pregnant. Furthermore, it was not possible to adjust for previous adverse pregnancy outcomes, nor did we have the statistical power to use permanent night workers only (*n* = 45) as a comparison group. As previously mentioned, we observed that night workers might have been slightly healthier than day workers, as they had a higher SEP (medium and high SEP: 93.3% vs. 81.1%). This difference was partly, because 17.5% of night workers were physicians compared to only 7.6% of day workers. However, we adjusted all analyses for SEP and other relevant confounders to account for these differences.

Another possible explanation for our null findings is the ‘healthy worker survivor effect’ (HWSE), where healthier workers are more likely to remain in their jobs (particularly night work in our study), while less healthier workers leave their work or change work schedule (particularly into day work in our study).^28^ This phenomenon is common in occupational studies and can lead to an attenuation of risk estimates of potential exposures.^29^ In our study, women who worked ≥25 night shifts had an OR slightly below 1 (OR = 0.90, 95% CI 0.61–1.31), which—if not a random finding—could be attributed to HWSE resulting in bias toward the null.

Begtrup et al. pointed out that of the 524 night workers (14% in the cohort) working ≥ 8 night shifts during gestational weeks 1–8 discontinued with night shifts after gestational week 13.^21^ The study was also based on data from DWHD, the same cohort as we used in our study. This may be because many women wait until the end of the first trimester to inform their employer about their pregnancy, given the heightened risk of miscarriage during this period.^35^ Although we did not examined self‐selection out of night work during pregnancy in our study, we assume this to be a minor issue, particularly in the analyses restricted to the first trimester.

As stated earlier, the sensitive time window for testicular descent varies in the literature (e.g., gestational weeks 1–12, 8–14, or 13–16), depending on whether the study subjects were animals or humans.^8^, ^9^, ^10^ This makes it challenging to investigate specific sensitive time windows for environmental exposures on the risk of cryptorchidism. However, to test the fact that testicular descent seems to be sensitive to exposures in specific time windows, the sensitivity analyses were restricted to either night shift work during the first trimester (gestational weeks 1–12) or second trimester (gestational weeks 13–22). This approach was based on evidence suggesting that maternal hormone levels during early pregnancy are critical for the testicular descent in male offspring.^5^ Thus, the trimester‐specific sensitivity analyses might have led to insufficient group size to produce meaningful results. Therefore, larger cohort studies are required to explore potential trimester‐specific associations.

Rate studies refer to ‘the male programming window’ occurring in gestational weeks 8–14 as an essential period for testicular descent.^8^, ^30^ Ideally, we would also have investigated exposure to night work in gestational weeks 8–14 in relation to the risk of giving birth to a male offspring with cryptorchidism. However, the number of cryptorchidism cases to mothers exposed in this specific period was too small to produce meaningful results.

In Denmark, the initial cryptorchidism diagnoses are made by hospital physicians at birth. However, this condition can also be diagnosed years later.^31^ We included both diagnoses of cryptorchidism identified at birth and offspring who underwent surgery later in childhood. Spontaneous testicular descent is common during the first year of life, which means that our definition may include transient cases of undescended testes. Due to the limitation in our sample size, we were unable to restrict the analyses to only male offspring with severe cryptorchidism requiring surgery, which might have provided a more valid outcome measurement.

Other limitations should also be considered. We were able to adjust for several factors, yet residual confounding or confounding from unknown factors cannot be ruled out. For instance, paternal factors and genetics have been associated with cryptorchidism.^32^ Having a father or an older brother with the condition increases the risk of cryptorchidism.^33^ However, these factors are unlikely to be related to women's night work. Moreover, in a study on concordance rates of cryptorchidism in twins, half‐ and full‐brothers, Jensen et al. found that the intrauterine environment plays a greater role in the development of cryptorchidism than genetic factors.^1^

Male infants born with cryptorchidism have been reported to have significantly lower birth weight, smaller chest circumstance, and lower placental weight at birth.^11^, ^34^ This is supported by the fact that cryptorchidism is common in preterm boys born before 37 weeks of gestation.^35^ We were unable to include data on birth weight or placental weight in our analysis. But low birth weight and preterm birth might not be strong intermediate factors for cryptorchidism according to the findings in Jensen et al.^1^ We consider this to be a minor issue.

Maternal diabetes may be a risk factor for cryptorchidism,^36^ and therefore, a potential confounder. However, the low number of women with maternal diabetes (*n* = 17) prevented us from adjusting for it in our models. Additionally, we lacked information on whether women were able to sleep during on‐call shifts or night shifts. This could be an important factor, if the mechanism behind cryptorchidism involves maternal exposure to light at night during pregnancy consequently disrupting melatonin production, which plays an important role in normal testicular descent.^33^ The inability to adjust for sleep during night shifts could potentially lead to misclassification because the women might maintain a normal circadian rhythm during sleep, which could result in an underestimation of the risk of cryptorchidism.

Finally, our study population was based on a nationwide cohort. It primarily consisted of healthcare professionals (nurses 45%), who may engage in more health‐promoting behaviors and have a healthier lifestyle compared to the general Danish population.^37^ This was indicated in our population, as we found a high proportion of women with a normal weight (65%) and non‐smokers (94%). Therefore, our findings may not be generalizable to pregnant women working night shifts in occupations outside the healthcare sector.

## 5 Conclusion

We found no association between any of the four dimensions of night work during pregnancy and the risk of having a male offspring with cryptorchidism. However, we cannot exclude the possibility of an association. The null findings may be attributed to the high number of healthy pregnant women working night shifts in this cohort or the limited extent of night shift work. Future studies would benefit from investigating similar exposure and outcome in a larger population with a broader range of occupations with night work, rather than focusing only on healthcare professionals working in the healthcare sector.

## AUTHOR CONTRIBUTIONS

Charlotte Bertelsen, Camilla Sandal Sejbæk, Luise Mølenberg Begtrup, Jens Peter Ellekilde Bonde, Paula E. C. Hammer, Ina Olmer Specht, and Esben Flachs Meulengracht conceived and designed the study. Anne Helene Garde and Johnni Hansen established and provided data from the DWHD. Charlotte Bertelsen, Camilla Sandal Sejbæk, Luise Mølenberg Begtrup, Paula E. C. Hammer, and Esben Flachs Meulengracht analyzed the data and Esben Flachs Meulengracht gave statistical support. Charlotte Bertelsen drafted the manuscript, and all authors interpreted the data and revised the manuscript. All authors are accountable for all aspects of the work.

## CONFLICT OF INTEREST STATEMENT

The authors declare no conflicts of interest.

## Supporting information



Supporting information
