# Supplementary material for: Night work during pregnancy and risk of cryptorchidism among male offspring: A Danish nationwide register‐based cohort study
Source: Andrology. 2025 May 7;14(1):140–8. doi: 10.1111/andr.70055 (PMC12670478; doi:10.1111/andr.70055)
Supplement: Supplementary file 1 — Supporting information [file ANDR-14-140-s001.docx]

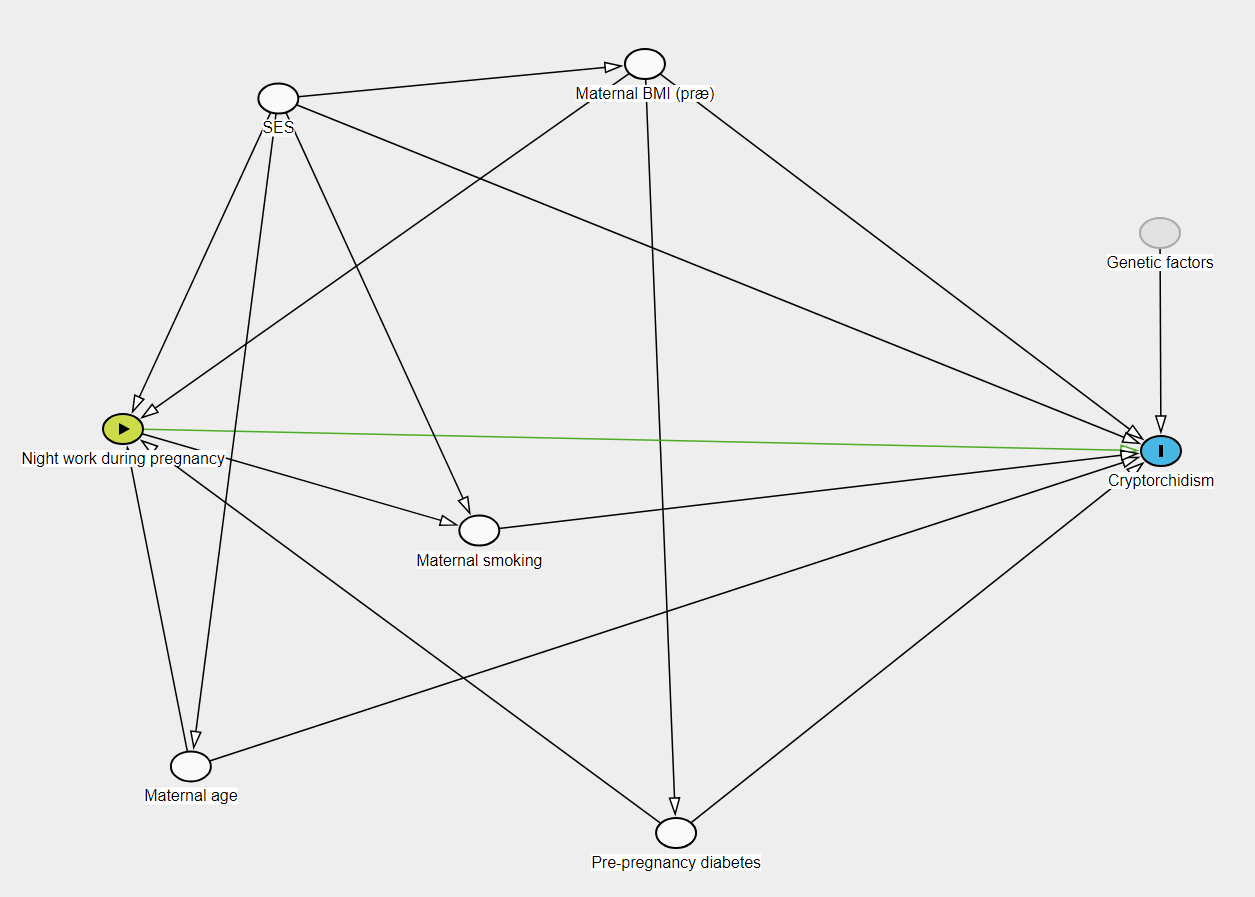
 **Supplementary Figure S1: Simplified DAG with confounders identified based on the previous literature. The identified confounders are included in our adjusted model are indicated with white circles.**
